# 2,3,7,8-Tetrachlorodibenzo-*p*-dioxin Slows the Progression of Experimental Cutaneous Leishmaniasis in Susceptible BALB/c and SCID Mice

**DOI:** 10.1371/journal.pone.0076259

**Published:** 2013-10-01

**Authors:** Gregory K. DeKrey, Riane E. Teagarden, Jerica L. Lenberg, Richard G. Titus

**Affiliations:** 1 School of Biological Sciences, College of Natural and Health Sciences, University of Northern Colorado, Greeley, Colorado, United States of America; 2 Department of Microbiology, Immunology and Pathology, College of Veterinary and Biomedical Sciences, Colorado State University, Fort Collins, Colorado, United States of America; Nihon University School of Medicine, Japan

## Abstract

In a model of experimental cutaneous leishmaniasis, pre-exposure of *Leishmania major*-resistant mice to 2,3,7,8-tetrachlorodibenzo-*p*-dioxin (TCDD), an aryl hydrocarbon receptor agonist, causes suppression of the protective anti-parasite T helper 1 response while paradoxically also reducing parasite burdens in those animals. In this study, we examined if TCDD exposure could also reduce parasite burdens in *L. major*-susceptible BALB/c mice. In the highest dose group (160 µg/Kg), TCDD treatment caused a significant reduction of parasite burdens by 10-fold after three weeks while also causing a significant lymphoid atrophy indicating suppression of the non-protective T helper 2 response. A dose-dependent delay of foot lesion progression was also observed such that lesion size in the highest dose group was less than half that of controls after 35 days of infection. Importantly, although TCDD exposure initially reduced disease severity and prolonged the course of disease by as much as three fold in some animals, this effect was transitory and TCDD did not induce resistance to *L. major* infection. Because TCDD exposure reduced *L. major* burdens in both resistant and susceptible mice, we hypothesized that TCDD reduces *L. major* burdens in mice by a mechanism that does not involve adaptive immunity. To test this, severe combined immunodeficient (SCID) mice were used. In mice infected with a moderate number of *L. major* (10,000), TCDD treatment caused a time- and dose-dependent decrease of parasite burdens by nearly 100-fold after six weeks in the highest dose group (200 µg/Kg). A significant and dose-dependent delay of foot lesion progression was also observed in these animals. These results indicate that TCDD exposure can reduce the severity of leishmanial disease in mice independent of adaptive immunity.

## Introduction

Leishmaniasis is a disease caused by infection with protozoan parasites of the genus *Leishmania* . Human leishmaniasis is found most commonly in tropical and subtropical regions of the world, and approximately two million cases are reported each year [1]. Natural infection occurs during a blood feeding event by an infected phlebotomine sand fly that will deliver *Leishmania* promastigotes into the skin of a potential host. Uptake of promastigotes by phagocytic cells can provide a suitable environment for transformation of promastigotes into the amastigote form that is most well adapted to the intracellular environment of the ultimate host cell, the macrophage. Depending on the species of *Leishmania* and the host’s response, disease can present in various forms from isolated cutaneous lesions to disseminated visceral pathology [2-5]. Experimental subcutaneous injection of *Leishmania* into mice can cause disease that mimics many aspects of a natural infection.

The use of mice infected with *Leishmania* (*L. major* in particular) was instrumental in defining the role for CD4+ T cells in resistance to *Leishmania* infection. In the absence of CD4+ T cells (e.g., in severe combined immunodeficient [SCID] mice), *L. major* infections are uncontrolled and extensive disseminated disease results [6,7]. However, with intact adaptive immune systems, most mouse strains (e.g., C57Bl/6, CBA, C3H) develop lesions at the site of *L. major* infection which ultimately resolve without significant dissemination. This resistant response is associated with T helper (Th)1-supported killing of *L. major* organisms by their host macrophages. In contrast, *L. major* infections in BALB/c mice do not resolve because of their predominating Th2 responses which are less supportive of *L. major* killing. The study of cutaneous leishmaniasis in mice infected with *L. major* was central to defining the Th1/Th2 paradigm [8-13].

The aryl hydrocarbon receptor (AhR) is a ligand-activated transcription factor of the Per-Arnt-Sim (PAS) family of proteins. Numerous exogenous and endogenous agonists of the AhR have been identified including such molecules as 6-formylindolo[3,2-b]carbazole (FICZ), lipoxin A4, and 2,3,7,8-tetrachlorodibenzo-*p*-dioxin (TCDD). TCDD is the most potent AhR ligand [14]. Because TCDD is both lipophilic and resistant to metabolic degradation, it has a long biological half-life (approximately 10 days in mice and 10 years in humans) which leads to prolonged AhR activation [15]. The AhR is expressed ubiquitously, but levels of expression vary. Within the immune system, AhR expression levels depend upon the cell type, location, and developmental state. Frericks et al. [16] reported the highest AhR expression in immature B cells and splenic dendritic cells, intermediate to high AhR expression in thymocytes, pancreatic T regulatory (Treg) cells and peritoneal macrophages, but low AhR expression in bone marrow macrophages and some T cell lines. Veldhoen et al. [17] reported that, among in vitro differentiated CD4+ T cells, AhR expression was highest in Th17 cells. In cells that express the AhR, agonist binding permits the AhR to form a heterodimer with the AhR nuclear translocator (ARNT). Translocation of AhR/ARNT into the nucleus is followed by binding to dioxin response elements (DREs) in the promotor regions of target genes and regulation of transcription [18]. Since the discovery of the AhR/ARNT signaling pathway, a large number of genes have been found to contain DREs including a number of genes related to immune function [19]. More recently, an AhR-dependent signaling pathway that does not involve ARNT or DREs has been described and termed the nongenomic AhR pathway [20]. Activation of this pathway leads to rapidly increased intracellular Ca^2+^ concentrations followed by activation of cytosolic phospholipase A2, production of arachidonic acid, activation of Src kinase, and upregulated expression of inflammatory markers such as cyclooxygenase-2.

Suppression of T cell immune function following TCDD exposure has been observed in nearly every animal model examined over the past four decades [21-24]. A potential mechanism to explain suppressed T cell responses has been suggested by recent studies showing that TCDD exposure enhances the number and function of Treg cells following antigen challenge [25,26]. A previous study in this laboratory [27] demonstrated suppressed adaptive immunity in TCDD-treated mice infected with *L. major*. Paradoxically, this study also found that TCDD exposure reduced parasite numbers in the lesions of *L. major*-infected C57Bl/6 (resistant) mice. Because a reduction of parasite burdens in resistant mice would most reasonably be associated with enhanced anti-*L. major* immunity, rather than suppression, we hypothesized that TCDD exposure reduces *L. major* burdens in mice by a mechanism that does not involve adaptive immunity. In the present study, we show that *L. major* burdens were reduced by exposure to TCDD in both BALB/c wild type and SCID mice. These findings suggest that TCDD exposure is detrimental to *L. major* survival in mice independent of its effects on adaptive immunity.

## Materials and Methods

### Ethics statement

This study was carried out in strict accordance with the recommendations in the Guide for the Care and Use of Laboratory Animals of the National Institutes of Health. The protocol was approved by the Institutional Animal Care and Use Committee of Colorado State University (99-113A). All efforts were made to minimize suffering.

### Animals and general animal procedures

Female BALB/c mice (BALB/cAnNCr or BALB/cByJ) and female SCID mice of the BALB/c background (C.B-17 scid/scid, a generous gift from R. Akkina) were maintained at the Laboratory Animal Resources facility, Colorado State University, on 12-hour light/dark cycles. BALB/c mice express a high affinity allele of the AhR (*Ahb-2*) and are considered TCDD responsive [28]. Within individual experiments, animals from a single source were used exclusively. Cellu-Dry bedding (Shepard Specialty Papers, Kalamazoo, MI) was used, and animals received autoclaved tap water and food (Harlan Sprague Dawley, Rodent Maintenance 8640) ad libitum. After infection with parasites, the thickness of foot lesions (defined as the thickness of the infected foot minus the thickness of the uninfected contralateral foot) was measured with a vernier caliper. Parasite burdens in foot lesions were determined by limiting dilution analysis as previously described [29]. Euthanasia was performed with an overdose of CO_2_.

### 2,3,7,8-tetrachlorodibenzo-*p*-dioxin (TCDD) and animal treatment

Crystalline TCDD (99% pure) was obtained from Cambridge Isotope Laboratories, Inc. (Andover, MA), dissolved in acetone, and mixed with peanut oil (Planters, Nabisco Brands Inc.). The acetone was driven off each solution using a stream of nitrogen, and dilutions were made in peanut oil as needed. Control solutions of peanut oil were prepared in a similar manner but without TCDD. The concentration of TCDD in each solution was confirmed by gas chromatography using a modified method of Laberton et al. [30]. Animals were treated with peanut oil (vehicle) or TCDD at various doses by gavage using volumes of 0.01 mL/g body weight.

### Parasites and animal infection

*Leishmania major*, strain LV39 (RHO/SU/59/P, Neal, or P strain), were maintained by biweekly passage through resistant mice followed by re-isolation from foot lesions as described previously [31,32]. One day after vehicle or TCDD treatment, all mice were anesthetized and injected with stationary-phase promastigote parasites into a single rear footpad each as described previously [27].

### Flow cytometry

Phenotype analysis of lesion-draining lymph node cells (popliteal plus inguinal) was performed using a XL flow cytometer (Coulter Corp.) as described previously [33,34]. Fluorochrome-conjugated monoclonal antibodies were purchased from Pharmingen (BD Biosciences): FITC anti-mouse CD45R (B220), FITC anti-mouse CD8, CyChrome anti-mouse CD4, PE anti-mouse CD25, and appropriate isotype-specific controls.

### Statistical analysis

Comparisons of lesion size and parasite burden were performed by 2-way analysis of variance followed by Tukey all-ways comparison post-hoc *t* tests. All tests were performed using SigmaPlot software (Systat Software, Inc.). Differences were considered significant at *p* < 0.05.

## Results

### Lesion size, parasite burdens, and lymph node cell phenotypes in wild type BALB/c mice

**Figure 1:**
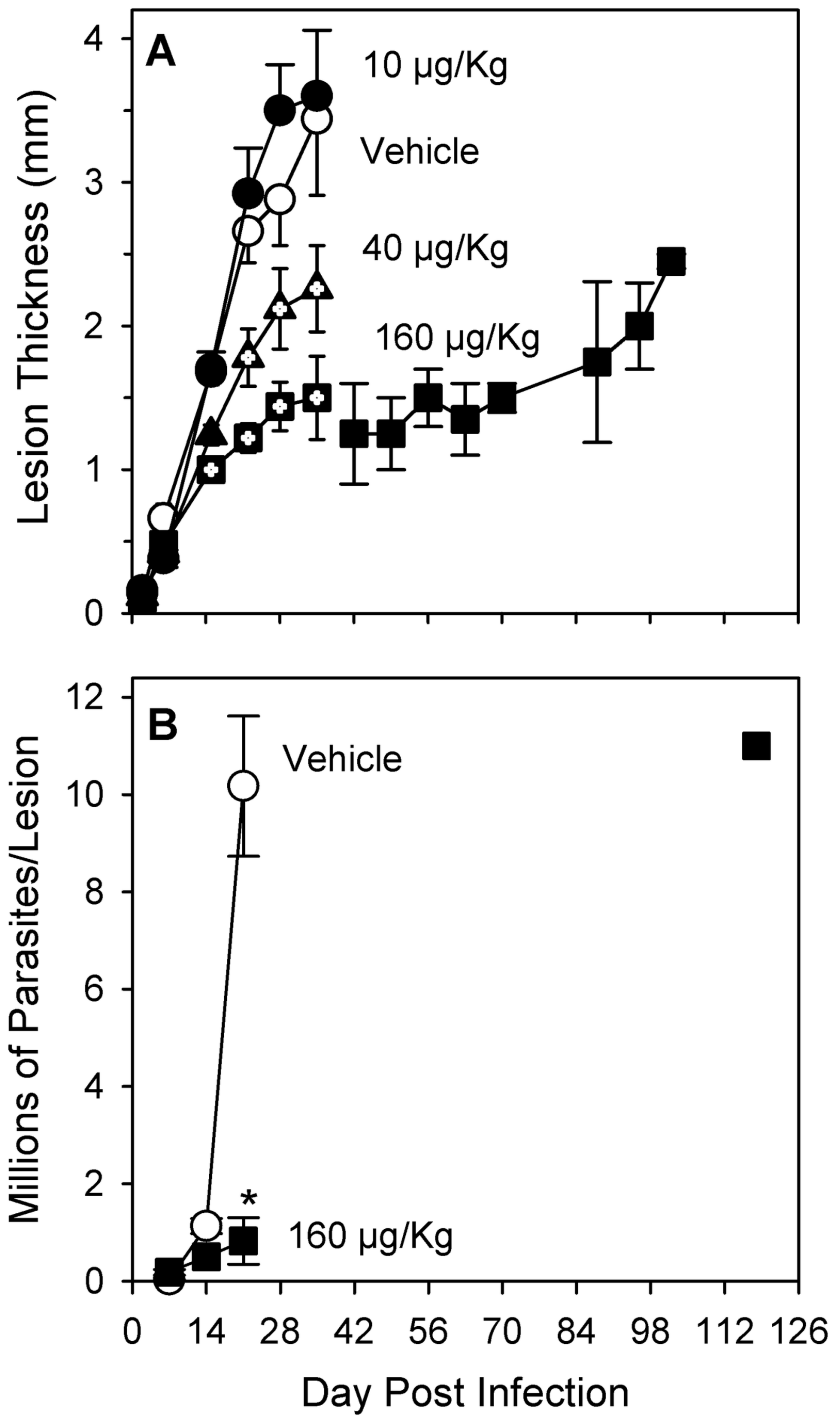
TCDD reduces parasite burdens and slows the progression of cutaneous leishmaniasis in BALB/c mice. Female BALB/c mice were treated with peanut oil (vehicle) or TCDD at various doses (per os) one day prior to infection with one million stationary phase L. major promastigotes in one rear footpad. Data are shown for 3-5 mice per treatment group on days 2-35 and are representative of three independent experiments. Data for two mice from one experiment are shown after day 35. **(A)** Lesion size is shown as mean ± SEM. Symbols with internal plus marks (+) indicate a statistically significant difference from vehicle-treated mice on that day (p < 0.05). **(B)** Parasite burdens in infected feet are shown (mean ± SEM) for mice euthanized on the days indicated: three mice per group up to day 35; after day 35, two mice were pooled (n = 1) . *Indicates a statistically significant difference from vehicle-treated mice on that day (p < 0.02).

After infection with *L. major*, the lesions of vehicle-treated (control) BALB/c mice developed rapidly and increased in thickness to a maximum of approximately 3.5 mm by day 35 (Figure 1A). An increase in intralesional *L. major* parasites was observed over time after infection in control mice, as expected [35], to a level of approximately 10 million at three weeks (Figure 1B). After day 35, because the lesions of control animals had ulcerated, they were euthanized to prevent any suffering that might result from further progression of lesion pathology. No significant differences in lesion size or pathology were observed at any time between control mice and mice treated with TCDD at the lowest dose of 10 µg/Kg (Figure 1A). In contrast, the lesions of mice in the intermediate (40 µg/Kg) and highest (160 µg/Kg) dose groups progressed in size more slowly than for control animals with a significant (*p* < 0.05) and dose-dependent reduction of lesion size to 2.3 mm and 1.5 mm thickness (66% and 44% of control), respectively, by day 35 post infection. The progression of lesion pathology was reduced such that lesion ulceration was not observed in the 40 µg/Kg and 160 µg/Kg dose groups until seven and fourteen days later than was observed in control mice, respectively. In one experiment, the lesions of two out of five mice in the highest dose group (data shown after day 35 in Figure 1A) did not ulcerate during 102 days of observation. The number of viable *L. major* organisms in the lesions of mice treated with TCDD at 160 µg/Kg were found to be lower, relative to control, in a time-dependent manner with a 56% reduction on day 14 (not significant) and a significant (*p* < 0.02) 10-fold reduction on day 21 post infection (Figure 1B). In this highest dose group, the two animals with the most delayed lesion progression ultimately did develop pathology (ulceration) sufficient to necessitate euthanasia. On day 118 post infection, parasite burdens in these mice were found to be 1.1 x 10^7^ per foot, a number equivalent to that seen in control mice on day 21 post infection. Inhibited disease progression in the 160 µg/Kg dose group was accompanied by a loss of body weight by day 26 post infection that was significantly different from the normal gain of body weight observed in control animals (Table 1). In addition, relative to control animals, mice in the highest dose group displayed a number of significant hallmark signs of TCDD toxicity: by day 26 post infection, thymus weights (normalized to body weights) were reduced by 57%, the number of lesion draining lymph node cells was reduced by 95%, the percentage of CD4+ cells in lesion-draining lymph nodes was reduced by 44%, and the percentage of those CD4+ cells expressing CD25 was increased nearly two-fold (Table 1).

**Table 1:** TCDD treatment decreased body weight and lymph node cell numbers 26 days after *L. major* infection.

	TCDD Treatment		
	Vehicle	160 µg/Kg	*p*
Body weight change (g)	2.23 ± 0.64	-0.23 ± 0.32	0.03
Thymus/Body weight (mg/g)	2.11 ± 0.36	0.91 ± 0.07	0.03
Total lymph node cells (x 10^-6^)	108.6 ± 21.4	5.3 ± 0.8	0.001
Percent CD4+	28.3 ± 2.3	18.7 ± 3.8	
Percent CD4+CD25+	12.3 ± 0.9	23.0 ± 3.2	0.03
Percent CD8+	13.0 ± 1.0	9.7 ± 1.9	
Percent CD45R+	54.7 ± 2.3	58.0 ± 3.5	

Female wild type BALB/c mice were treated with peanut oil (vehicle) or TCDD (160 µg/Kg per os) one day prior to infection with one million stationary phase *L. major* promastigotes in one rear footpad. Data represent mean ± SEM for three animals per treatment group.

### Lesion size and parasite burdens in SCID mice

**Figure 2:**
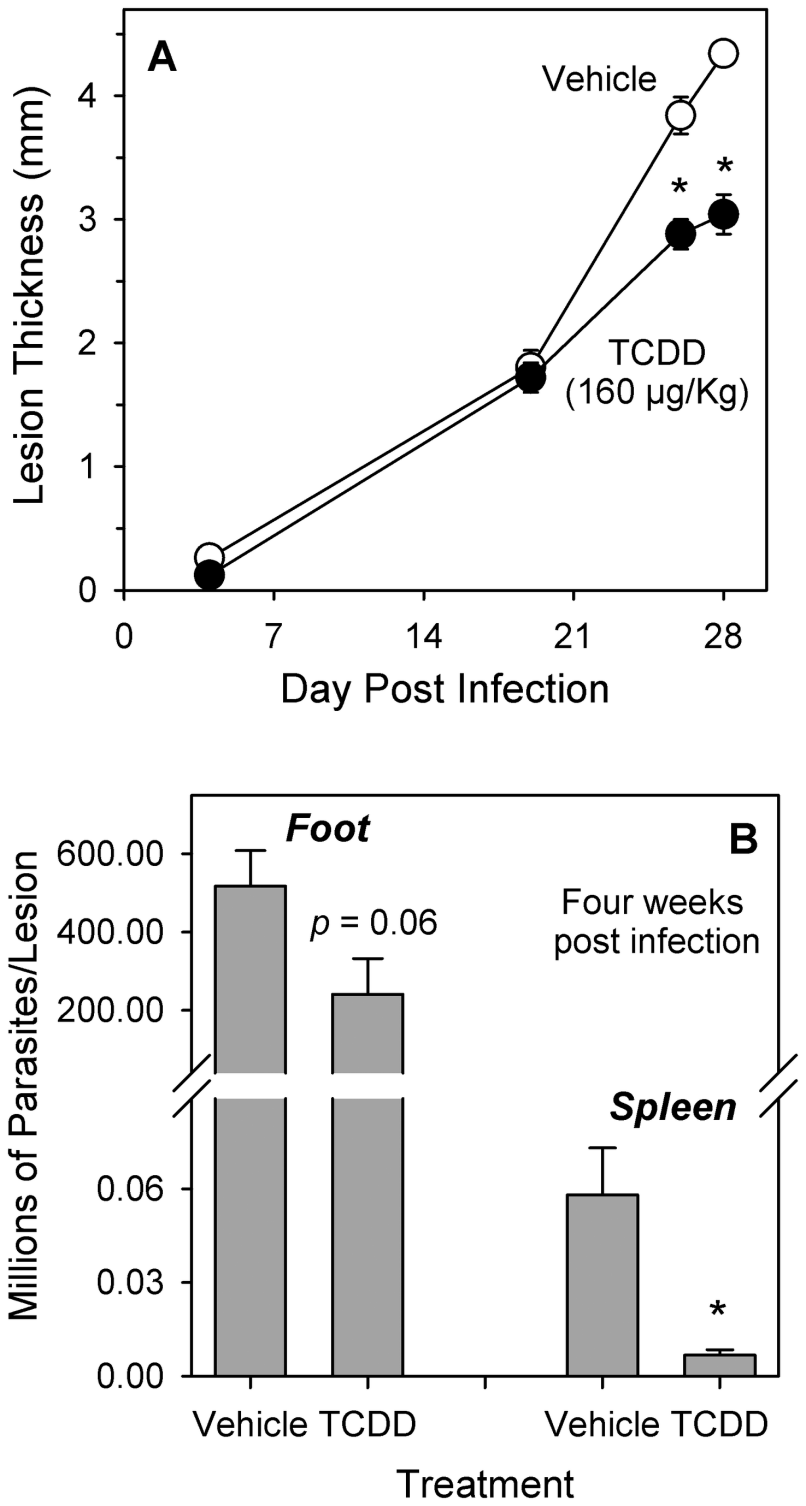
TCDD reduces parasite burdens and slows the progression of cutaneous leishmaniasis in SCID mice. Female SCID mice were treated with peanut oil (vehicle) or TCDD (160 µg/Kg body weight) per os one day prior to infection with one million stationary phase L. major promastigotes in one rear footpad. **(A)** Lesion size is shown as mean ± SEM for five mice per group. *Indicates a statistically significant difference from vehicle-treated mice on that day (p < 0.05). The results are representative of four separate experiments. **(B)** Parasite burdens in individual infected feet were analyzed at four weeks post infection (six mice per treatment group; data represent mean ± SEM). Parasite burdens in individual spleens were analyzed at four weeks post infection (three mice per treatment group; data represent mean ± SEM). *Indicates a statistically significant difference from vehicle-treated mice on that day (p < 0.05). The results are representative of 2-3 separate experiments.

**Figure 3:**
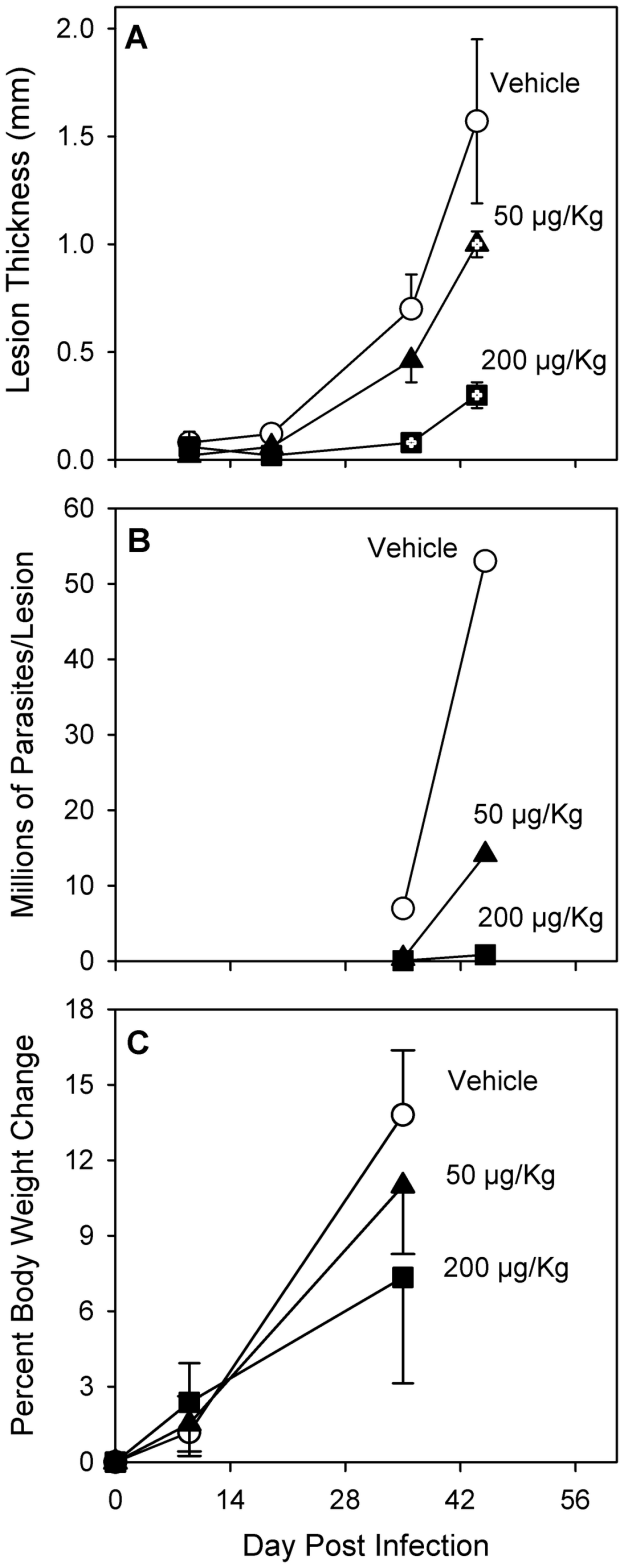
Effects of TCDD in SCID mice after low dose infection. Female SCID mice were treated with peanut oil (vehicle) or TCDD at various doses (per os) one day prior to infection with ten thousand stationary phase *L. major* promastigotes in one rear footpad. (**A**) Lesion size is shown as mean ± SEM for 3-5 mice per time point. Symbols with internal plus marks (+) indicate a statistically significant difference from vehicle mice on that day (*p* < 0.05). All animals within a treatment group were euthanized following the last indicated measurement. (**B**) Mice were euthanized on the days indicated, and the infected feet of 2-3 mice per group were analyzed as a pool (n=1) for parasite burdens. (**C**) Percent body weight change is shown as mean ± SEM for 5 mice per time point. Data shown on day 35 reflects body weight change of animals measured either on day 35 or day 37.

After infection with 1 x 10^6^ *L. major*, the lesions of vehicle-treated (control) SCID mice developed rapidly and increased to a thickness greater than 4 mm by day 28 (Figure 2A). The number of viable parasites in the infected feet of control SCID mice was greater than 5 x 10^8^ after four weeks, and, at that same time, dissemination of parasites away from the site of infection was indicated by the presence of parasites in the spleens of those mice (Figure 2B). Lesion thickness for TCDD-treated SCID mice (160 µg/Kg) increased in parallel to that of control SCID mice with no differences through day 19. Thereafter, significantly smaller lesion size (*p* < 0.05) was observed through day 28 at which point the size of lesions in TCDD-treated SCID mice was 70% of that for controls (Figure 2A). Treatment of SCID mice with TCDD resulted in a 53% reduction (not significant) in the number of parasites per infected foot after four weeks, and the number of parasites in the spleens of TCDD-treated SCID mice was significantly reduced (*p* < 0.05) by 88% (Figure 2B). Because of the large number of parasites found in the foot lesions of SCID mice after injection of 1 x 10^6^ *Leishmania* organisms, and because all SCID mice had lost approximately 15% of body weight by day 28 of infection with no significant difference between control and TCDD-treated animals (data not shown), the number of stationary-phase *L. major* promastigotes injected per foot was reduced 100-fold to 1 x 10^4^ in subsequent experiments with SCID mice. As shown in Figure 3, injection of fewer parasites resulted in slower progression of disease but no loss of body weight. In vehicle-treated (control) SCID mice, the size of foot lesions reached 1.5 mm by the sixth week post infection, and the number of viable parasites was found to be 53 x 10^6^ per foot at that time (Figure 3A-3B). Treatment of SCID mice with TCDD resulted in a significant delay (*p* < 0.05) of lesion development that was dose-dependent such that, by six weeks post infection, mice treated with TCDD at 50 µg/Kg and 200 µg/Kg had lesions that were 36% and 91% smaller than control mice, respectively (Figure 3A). Parasite burdens in TCDD-treated mice were also reduced in a time- and dose-dependent manner such that, after six weeks of infection, the number of viable parasites in mice treated with TCDD at 50 µg/Kg and 200 µg/Kg was 27% and 2% of that found in control mice (Figure 3B). Body weights of control mice increased approximately 14% through day 37 post infection (Figure 3C). TCDD treatment had no statistically significant effect on body weight change over the observed time-frame, although an approximate 50% reduction of body weight increase was suggested on day 37 in the highest dose group.

## Discussion

In this study, exposure to TCDD one day prior to infection with *L. major* resulted in two principle findings in both wild type BALB/c and SCID mice: 1) a dose-dependent decrease of lesion size resulting in delayed disease progression, and 2) a reduction of parasite burdens that appeared three weeks or more after infection. A definitive mechanism to explain these finding has yet to be determined. Importantly, unlike for *Streptococcus pneumoniae* infection [36,37], exposure to TCDD did not appear to change the ultimate outcome of *L. major* infection. Although it is possible that the TCDD-mediated changes in disease progression presented here are the result of direct toxicity on *L. major* itself, this possibility is considered to be unlikely. One reason is that studies in this laboratory have found no changes in promastigote parasite proliferation or infectivity when exposed to TCDD at concentrations up to 0.5 x 10^-7^ M in vitro (data not shown). Moreover, phylogenetic studies have identified orthologues of vertebrate AhR proteins in multiple invertebrate species; but, unlike their vertebrate counterparts, these invertebrate proteins do not appear to bind molecules recognized as AhR agonists [38].

In wild type BALB/c mice, one explanation for the TCDD-mediated reduction of lesion size could be the underlying suppression of the anti-*L. major* T cell response. A Th1 response that supports killing of *L. major* by infected macrophages is key to resistance, but TCDD suppresses that response (Table 1) [27]. Suppression of T cell responses using anti-CD4 monoclonal antibody was shown by Liew et al. [39] to also decrease lesion size in *L. major*-infected BALB/c mice. Reduced inflammatory pathology following TCDD exposure at cumulative doses similar to those used in the present study has been reported in other disease models using C57Bl/6 mice such as herpes virus infection [40] and experimental colitis [41], and this reduced inflammation was correlated with increased Treg/T effector cell ratios. A significant increase in the percentage of CD25+ cells among the CD4+ population of lesion-draining lymph node cells was observed in TCDD-treated *L. major*-infected mice (Table 1), but a specific impact on Treg cells was not examined. This leaves open the possibility of Treg cells playing a role in reducing lesion size in this model, at least in wild type BALB/c mice. However, an altered Treg/T effector cell ratio could not explain the observed reduction of leishmanial lesion size in TCDD-treated SCID mice which are deficient of T cells (Figures 2 & 3). Because a parallel reduction of parasite burdens was observed in both wild type and SCID mice, we conclude that reduced parasite burdens and delayed disease progression are effects of TCDD exposure that are, at least in part, independent of its effects on adaptive immunity.

Following infection of mice with *L. major*, significant differences in cellular infiltrates in lesions have been observed between resistant and susceptible strains, particularly with regard to MRP14-expressing cells [42]. MRP14 (S100A9) is a member of the S100 calcium binding family of proteins and is a marker for a type of inflammatory macrophages [43]. MRP14+ macrophages have been shown to take up *L. major*, but they are less active at killing intracellular *Leishmania* than F4/80+ macrophages, which predominate in the lesions of resistant mice, and they are less sensitive than F4/80+ macrophages to interferon-γ-stimulated activation and killing of *Leishmania* [44]. It has been suggested that MRP14+ macrophages are the predominant host cell leading to survival of *L. major* in vivo [45]. Sunderkotter et al. [42] showed that the number of MRP14+ cells infiltrating lesions of *L. major*-infected susceptible mice was increased after one week, relative to resistant mice, and that this was likely due to the presence of T cells because no resistant/susceptible strain differences were observed when using athymic mice. Thus, increased disease severity in BALB/c mice correlated with an increase of MRP14+ cells in leishmanial lesions. Because of the profound T cell suppression observed in TCDD-treated wild type BALB/c mice in the present study, it is unlikely that a T cell-mediated increase of lesional MRP14+ cells occurred. Therefore, one explanation for the reduced lesion size and parasite burdens observed in TCDD-treated wild type BALB/c mice could be the failure of a T cell-mediated increase of MRP14+ macrophage infiltrates. However, this cannot be the sole explanation for this phenomenon because reduced disease severity was also observed in TCDD-treated SCID mice. Vogel et al. [46] found that TCDD treatment alone caused increased mRNA expression for chemokines KC and MCP-1 as well as for F4/80 in various tissues. They interpreted the latter observation to reflect an increased infiltration of F4/80^+^ cells into those tissues. An increase in F4/80^+^ cells infiltrating the leishmanial lesions of TCDD-treated mice could contribute to reduced parasite burdens in both wild type and SCID mice. Curiously, Temchura et al. [47] found that MRP14 expression was upregulated by in vivo TCDD exposure in some thymocytes, a finding that correlated with increased egress from the thymus. Whether or not TCDD also upregulates MRP14 expression in monocytes or macrophages has not been examined.

In a previous study in this laboratory, Bowers et al. [27] found that TCDD exposure at doses less than 40 µg/Kg had no significant effect on the course of disease in *L. major*-infected C57Bl/6 mice. Given the critical role of T cell responses in governing the outcome of leishmanial disease [8-13], and given the large number of studies demonstrating altered T cell responses in mice exposed to TCDD at doses lower than 40 µg/Kg [21-24], this finding could be considered unexpected. However, an earlier study by DeKrey and Kervkliet [48] found that TCDD at 40 µg/Kg was a threshold dose in C57Bl/6 mice for significant elevation of serum corticosterone levels, whereas significant suppression of an allograft rejection response in those same mice was evident at doses of TCDD as low as 5 µg/Kg. Corticosterone is the primary glucocorticoid produced in mice. Importantly, glucocorticoid treatment has been shown to reduce *L. major* burdens in mice. Steinbrink et al. [44] found that a single dose of dexamethasone (10 mg/Kg) one day prior to infection was sufficient to significantly reduce the number of *L. major* in foot lesions, lymph nodes and spleens of BALB/c mice beginning at 21 days of infection, observations that correlated with significantly reduced infiltration of MRP14+ cells into foot lesions. Moreover, they found that dexamethasone delayed disease progression such that lesion ulceration occurred two weeks later in treated mice than in control mice. These results are very similar to those presented here for TCDD-treated mice, and they suggest that reduced *L. major* burdens in TCDD-treated wild type BALB/c and SCID mice may be indirectly caused by an elevation of corticosterone. This possibility will be examined in future studies.

The effects of TCDD in mammals are nearly universally attributed to its action as an agonist of the AhR, and there is no evidence to the contrary presented in this study. A recent study by Elizondo et al. [49] examined the effect of AhR deficiency on *L. major* infection in C57Bl/6 mice and found significantly reduced parasite burdens in AhR knockout mice, relative to AhR^+/+^ mice, at eight weeks post infection. The size of leishmanial lesions in these AhR-deficient mice was significantly increased within the first three weeks after infection but significantly reduced after four weeks. These findings are interesting because either exposure to an AhR-activating ligand (TCDD, shown here) or the absence of functional AhR [49] had similar effects by decreasing *L. major* burdens and, ultimately, decreasing lesion size. Similar phenomena using different experimental models have been reported. Latchney et al. [50] found reduced hippocampal neurogenesis and function in both TCDD-treated mice and AhR deficient mice relative to untreated wild type mice. Veldhoen et al. [17] found that both AhR deficiency (using AhR-null mice) and AhR activation (using FICZ) led to reduced clinical pathology, relative to control wild type mice, in a model of experimental autoimmune encephalitis. Shi et al. [51] showed that expression of the AhR in mice was necessary for optimal resistance to *Listeria monocytogenes*, whereas other studies found that AhR activation (using TCDD) reduced resistance to this bacterium [52-54]. These findings underscore the subtle complexity of the AhR’s regulatory role in cellular physiology. Elizondo et al. [49] attributed the enhanced *L. major* resistance of AhR-null mice to, at least in part, the elevated levels of plasma tumor necrosis factor (TNF)-α observed in those mice. Indeed, enhanced TNF-α production may be a common mechanism leading to reduced parasite burdens in both AhR-null mice and TCDD-treated wild type mice. In wild type mice and in human cells (including macrophages), enhanced production of TNF-α and other proinflammatory factors has been observed following TCDD exposure, with or without other stimuli [55-60]. Phagocytosis of *Leishmania* by macrophages can enhance TNF-α production by those cells [61], and TNF-α has been clearly shown to enhance resistance to *Leishmania* in mice by upregulating nitric oxide production by infected macrophages [62-64]. The mechanism underlying elevated TNF-α levels was not determined in the study by Elizondo et al. [17]. One possible source could be macrophages. Alternatively, because of the reduced numbers of Foxp3+CD25+CD4+ Treg cells observed in AhR-null mice in that study, increased numbers of T effector cells might be expected as a source of additional TNF-α. Further study will be required to determine if a common mechanism for reduced *L. major* burdens exists for AhR-null and AhR-activated mice.

In the present study, TCDD treatment (160 µg/Kg) and *L. major* infection led to a significant loss of body weight in wild type BALB/c mice when measured after 26 days (Table 1) but no mortality was observed. BALB/c mice do not loose body weight over a 40 day period solely due to infection with *L. major*, although they do fail to gain weight relative to uninfected mice [65,66]. Loss of body weight and/or reduced body weight gain following high dose TCDD exposure (without pathogen challenge) has been reported in multiple species. Among inbred mice that express the high affinity aryl hydrocarbon receptor (AhR^b/b^), sensitivity to body weight loss varies with strain and between studies. For example, a National Toxicology Program study reported that male and female B6C3F1 mice displayed no loss of body weight or mortality after a single oral treatment with TCDD at 200 µg/Kg when compared to vehicle-treated control mice [67]. In contrast, Vos et al. found that male C57Bl/6 mice suffered significant body weight loss for three weeks after a single oral treatment with TCDD at 150 µg/Kg and also experienced a 93% mortality rate with a mean time to death of 22.6 days [68]. Clearly, the female wild type BALB/c mice in the present study suffered toxicity from TCDD exposure that was non-lethal. It should be noted that female BALB/c mice are less sensitive to some aspects of TCDD toxicity (e.g., hepatic porphyria) than are male BALB/c mice [69]. How *L. major* infection influenced TCDD’s effect on body weight is unclear and was not specifically addressed in this study. However, it is possible that the body weight loss observed in TCDD-treated wild type BALB/c mice may have been influenced by the adaptive anti-*Leishmania* immune response more than the *Leishmania* infection itself. This is suggested by the fact that SCID mice gained weight rather than losing weight after *L. major* infection and TCDD treatment at doses up to 200 µg/Kg (Figure 3). Although it is possible that the reduction of parasite burdens in TCDD-treated mice is caused by overt TCDD toxicity to the mouse, the lack of significant body weight loss in TCDD-treated SCID mice argues against this.

In conclusion, we show here that TCDD exposure causes a delay in the progression of disease in *L. major*-infected wild type and SCID mice. These results suggest that TCDD’s effects are mediated through a mechanism that, at least in part, does not involve adaptive immunity. Potential mechanisms to explain these observations include reduced infiltration of suitable host macrophages at the site of infection and/or enhanced inflammatory cytokine production with accompanying increased parasite killing.
